# Are Columnar Cell Lesions the Earliest Non-Obligate Precursor in the Low-Grade Breast Neoplasia Pathway?

**DOI:** 10.3390/curroncol29080447

**Published:** 2022-08-11

**Authors:** Sarah Strickland, Gulisa Turashvili

**Affiliations:** 1 Department of Pathology and Laboratory Medicine, Faculty of Medicine, University of Ottawa, Ottawa, ON K1N 6N5, Canada; 2 Department of Pathology and Laboratory Medicine, Emory University Hospital, Emory University School of Medicine, Atlanta, GA 30322, USA

**Keywords:** breast, columnar cell lesions, precursor, low-grade neoplasia, carcinogenesis

## Abstract

Columnar cell lesions (CCLs) of the breast comprise a spectrum of morphologic alterations of the terminal duct lobular unit involving variably dilated and enlarged acini lined by columnar epithelial cells. The World Health Organization currently classifies CCLs without atypia as columnar cell change (CCC) and columnar cell hyperplasia (CCH), whereas flat epithelial atypia (FEA) is a unifying term encompassing both CCC and CCH with cytologic atypia. CCLs have been increasingly recognized in stereotactic core needle biopsies (CNBs) performed for the assessment of calcifications. CCLs are believed to represent the earliest non-obligate precursor of low-grade invasive breast carcinomas as they share molecular alterations and often coexist with entities in the low-grade breast neoplasia pathway. Despite this association, however, the risk of progression of CCLs to invasive breast carcinoma appears low and may not exceed that of concurrent proliferative lesions. As the reported upgrade rates of pure CCL/FEA when identified as the most advanced high-risk lesion on CNB vary widely, the management of FEA diagnosed on CNB remains controversial. This review will include a historical overview of CCLs and will examine histologic diagnostic criteria, molecular alterations, prognosis and issues related to upgrade rates and clinical management.

## 1. Introduction

Columnar cell lesions (CCLs) of the breast comprise a spectrum of morphologic alterations of the terminal duct lobular unit (TDLU) involving variably dilated and enlarged acini lined by columnar epithelial cells. While CCLs have historically been described using a variety of terms, the World Health Organization currently classifies CCLs without atypia as columnar cell change (CCC) and columnar cell hyperplasia (CCH), whereas flat epithelial atypia (FEA) exhibits low-grade (monomorphic) cytologic atypia and may be used as a unifying term encompassing both CCC and CCH with atypia [1].

In part due to the implementation of widespread screening mammography, CCLs have been increasingly recognized in stereotactic core needle biopsies (CNB) performed for the assessment of calcifications [2,3,4,5]. CCLs have been postulated to represent the earliest non-obligate precursor of low-grade invasive breast carcinomas as they share molecular alterations and often coexist with entities in the low-grade breast neoplasia pathway, including atypical ductal hyperplasia (ADH), lobular neoplasia (LN), low-grade ductal carcinoma in situ (DCIS) and tubular carcinoma [6,7,8,9,10,11,12,13,14,15,16,17,18,19,20]. Despite this association, however, the risk of progression of CCLs to invasive breast carcinoma is low and may not exceed that of concurrent proliferative lesions. As the reported rates of pure CCL/FEA when identified as the most advanced high-risk lesion on CNB vary widely, the management of FEA diagnosed on CNB remains controversial. This review will include a historical overview of CCLs and will examine histologic diagnostic criteria, molecular alterations, prognosis and issues related to upgrade rates and clinical management. 

## 2. Historical Overview and Terminology

CCLs have long been recognized by pathologists but have been previously described using a wide assortment of names, including blunt duct adenosis [21], columnar alteration of lobules [6], columnar cell alterations with atypical snouts [6], atypical cystic lobules [7], atypical lobule of type A [22], hyperplastic enlarged lobular unit [23], unfolded lobules [24], monomorphous-type clinging carcinoma [25], and flat epithelial atypia without intraluminal proliferation, originally designated as ductal intraepithelial neoplasia 1b (DIN1b) [26] and later DIN1a [27]. CCLs with more complex architectural patterns such as micropapillary formations, rigid cellular bridges, bars and arcades or sieve-like fenestrations have also been previously categorized as CCH with moderate or severe atypia [16].

In 2003, Drs. S. Schnitt and A. Vincent-Salomon grouped CCLs into two categories-CCC and CCH, both of which should lack significant cytologic atypia. In contrast, the presence of high-grade nuclear features, even if present in a single cell layer, merits the designation of high-grade DCIS, while complex architectural patterns are best classified as ADH or low-grade DCIS depending on their extent [28]. In 2005, this classification was expanded into six groups by Simpson et al.: (1) CCC, (2) CCH, (3) CCH with architectural atypia, (4) CCH with cytologic atypia, (5) CCH with cytologic and architectural atypia, and (6) CCC with cytologic atypia. The authors noted difficulties in interobserver reproducibility encountered due to limitations in assessing mild nuclear pleomorphism [29]. In 2007, while investigating possible precursor lesions less than ADH or DCIS, Goldstein et al. defined monomorphic epithelial proliferations (MEPs) as a slightly overcrowded, predominantly single layer of monomorphic, luminal epithelial cells involving TDLUs in an overgrowth extension pattern [30]. MEPs tended to retain the architecture of the TDLU but could also expand the acini in an unfolded lobule-like pattern without associated complex architectural features. The cytomorphologic features of MEPs ranged from innocuous to slightly atypical and included three patterns: (1) columnar cells with uniform ovoid to elongated nuclei, (2) cuboidal cells with mildly enlarged round to oval nuclei, and (3) cuboidal to columnar cells with scant cytoplasm and round to slightly ellipsoid nuclei. The MEP entity included any lesion that was monomorphic and slightly hypercellular, regardless of the cytologic features, and was not restricted to lesions termed by others as CCLs or FEA. MEP lesions were significantly more frequent near initial breast resection margins in patients with clonally related local breast cancer recurrences than in patients with clonally distinct recurrences or no recurrences. Furthermore, some MEPs in clonal and distinct local recurrences shared allelic imbalance microsatellite markers with their respective initial carcinomas. Thus, MPEs appear to form the pool of partially transformed precursor lesions less than ADH or DCIS, similar to CCLs.

Blunt duct adenosis (BDA) was first described by Foote and Stewart [21], and has previously been considered a synonym of CCL without atypia [31,32]. Other authors, however, describe morphologic differences between BDA and CCL, including flattened branching configurations, irregular ducts, conspicuous myoepithelial cells with clear cytoplasm and slightly expanded, fibrotic and cellular intralobular stroma [33,34]. In the 2012 edition of the WHO classification of tumors of the breast, BDA is considered synonymous with CCC/CCH and is distinguished from FEA by the lack of cytologic atypia [35]; however, in the 2019 edition BDA is mentioned as “not recommended terminology” for CCLs [1]. De Boer et al. sought to assess molecular characteristics of low-grade breast neoplasia (whole-arm loss of chromosome 16q) using multiplex ligation-dependent probe amplification in a cohort of BDA and CCLs with and without atypia [36]. 16q loss was common in CCLs with and without atypia but was absent in BDA. In a subsequent publication, the authors stated that given the lack of 16q loss together with recognizable architectural and cytonuclear features, BDA is not a true precursor in the low-grade neoplasia family and should be considered as a separate entity from CCLs [37]. Follow-up data was also recommended to assess the reproducibility of the histologic diagnoses of BDA versus CCL and to determine if the risk of progression to breast cancer of BDA is that of other benign lesions. 

The current 5th edition of the WHO classification of tumors of the breast from 2019 classifies CCLs without atypia as CCC and CCH. FEA is the recommended term for CCLs with atypia, although acceptable terminology also includes CCC with atypia and CCH with atypia [1].

## 3. Radiologic Findings

CCLs are most frequently identified in CNBs performed for mammographically detected calcifications. While the majority of CCLs manifest as grouped amorphous calcifications, fine pleomorphic or punctate calcifications can also be observed [2,3,6,8,9,10,32,38,39,40,41,42,43,44,45,46,47,48,49]. The calcifications associated with CCLs may be indistinguishable from other causes of suspicious calcifications such as ADH or DCIS, necessitating biopsy or excision for diagnosis [2]. Less often FEA may appear as a mammographically detected mass, architectural distortion or asymmetry [10,41,42,43,44,46,47,49,50]. Some CCLs may not be detected due to their small size and lack of associated calcifications or other radiologic abnormalities. 

Sonographically, CCLs with and without atypia may present as cystic or solid lesions as well as a mass with irregular, microlobulated or indistinct borders and hypoechoic or complex echotexture [2,38,39,43,45,46]. There are no reported specific mammographic or ultrasound features which aid in the distinction of patients with CCLs with or without atypia [2,45].

Although there is a dearth of literature with regards to magnetic resonance imaging (MRI) features in CCLs, findings described include non-mass enhancement (NME) [38,51] and irregular clumped enhancement [46]. Santucci et al. describe a series of 139 patients with histologic borderline (B3) lesions, of which 31 (14.39%) were diagnosed as FEA [47]. Six patients in this cohort had MRI investigations, including five (83%) with post-contrast mass-like enhancement and one (17%) with segmental enhancement. The borders of the masses were described as regular, lobulated, irregular and with blurred edges. Kinetic curves were evenly split between types I, II and III. CCLs diagnosed purely as MRI findings with no mammographic or ultrasound correlates appear exceptionally rare [52,53,54,55,56].

Approximately 40% of women in breast screening programs have mammographically dense breasts. Percent mammographic density (PMD) of the breast reflects variations in the number of non-epithelial and epithelial cells, and collagen. Extensive PMD or elevated mammographic density (MBD) is associated with an increased risk of developing invasive carcinoma [57]. A possible association between CCLs and breast tissue composition was first reported in 2009 [58]. In a cohort of 236 randomly selected tissue samples obtained by bilateral subcutaneous mastectomy from a forensic autopsy series, CCLs were identified in 40 (17%) cases. The presence of CCL was associated with measures of breast tissue composition, such as high Faxitron Wolfe Density, high density estimated by percentage non-adipose tissue area, high percentage collagen, and high percentage glandular area. In a more recent study of 3400 women, after adjusting for age and body mass index, there was a positive association between CCH/FEA and high MBD (OR 1.3, 95% CI, 1.0–1.6) [59].

## 4. Histologic Features

**Figure 1:**
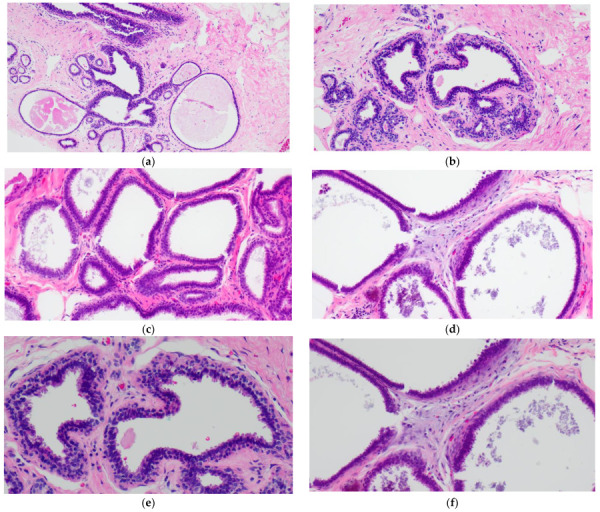
Columnar cell change (CCC) showing variably sized and shaped (**a**,**c**) acini lined by one to two layers of columnar-shaped epithelial cells with uniform, ovoid to elongated nuclei oriented perpendicular to the basement membrane; apical cytoplasmic snouts are seen at the luminal surface and an outer layer of myoepithelial cells is evident (**b**,**d**–**f**). There are luminal secretions (**a**,**c**,**d**) and calcifications (**d**,**f**).

The simplest form of CCL is CCC which is characterized by variably dilated acini with relatively smooth contours lined by one to two layers of columnar epithelial cells (Figure 1a,c). The lesional cells of CCC exhibit ovoid to elongated nuclei that are oriented perpendicular to the basement membrane in a regular fashion and contain evenly dispersed chromatin without conspicuous nucleoli (Figure 1b,d–f). Apical cytoplasmic blebs or snouts may be present at the luminal surface (Figure 1d–f), and mitotic figures are rarely encountered.

**Figure 2:**
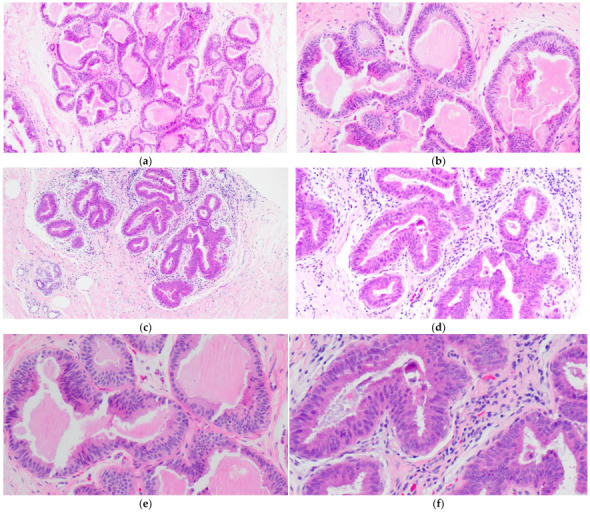
Columnar cell hyperplasia (CCH) showing variably dilated acini lined by more than two layers of columnar-shaped cells with (**a**,**c**,**e**,**f**) with nuclear crowding and overlapping, and formation of small tufts (**b**,**d**). There are luminal secretions (**a**,**b**,**e**) and calcifications (**c**,**d**,**f**).

CCH is composed of variably dilated acini lined by columnar cells with cytologic features similar to CCC but with cellular stratification of more than two cell layers (Figure 2a,c,e,f). Crowding or overlapping of the nuclei may give the appearance of nuclear hyperchromasia, and the proliferating cells may form small mounds, tufts or short micropapillations (Figure 2b,d). Both CCC and CCH can be associated with luminal secretions (Figure 1a,c,d and Figure 2a,b,e) and calcifications (Figure 1d,f and Figure 2c,d,f).

**Figure 3:**
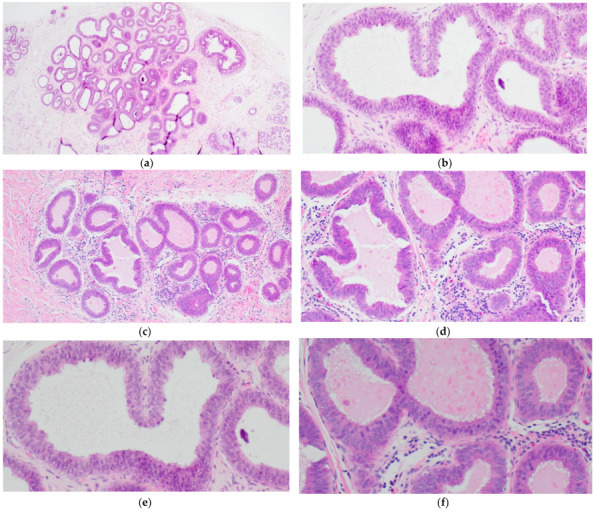
Flat epithelial atypia (FEA) composed of variably dilated acini (**a**,**c**) with calcifications (**a**,**b**) and low-grade cytologic atypia characterized by the presence of rounded or ovoid nuclei with loss of polarity, mildly increased nuclear to cytoplasmic ratio and prominent nucleoli in some cells (**b**,**d**–**f**).

**Figure 4:**
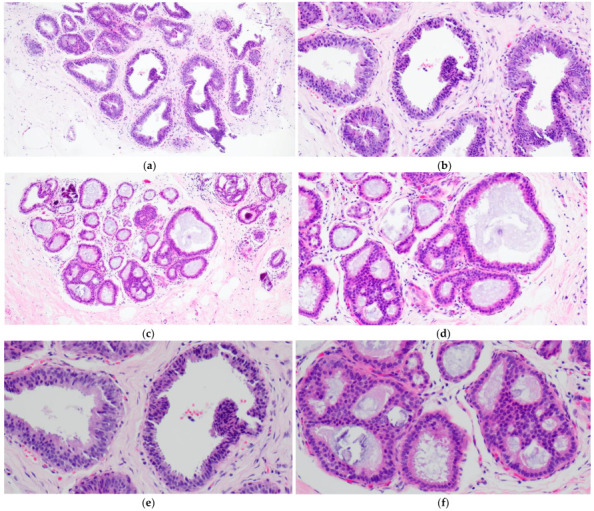
Columnar cell lesion (CCL) with foci of architectural complexity comprising focal formation of a micropapillary structure (**a**,**b**,**e**) and cribriforming with rigid cellular bridges (**c**,**d**,**f**) associated with low-grade cytologic atypia, consistent with atypical ductal hyperplasia.

FEA now encompasses the term for CCLs with low-grade (monomorphic) cytologic atypia resembling the nuclei of low-grade DCIS. FEA has architectural features of CCC or CCH (Figure 3a,c), but with superimposed cytologic atypia characterized by the presence of rounded or ovoid nuclei with loss of polarity, mildly increased nuclear to cytoplasmic ratio and variably prominent nucleoli (Figure 3b,d–f). Some cases of CCL may lack such classic morphologic features but the presence focal or patchy cytologic atypia may be sufficient to warrant the diagnosis of CCL with atypia. The latter is indeed a term preferred over FEA by some pathologists. Lesions with columnar cells but more complex architectural patterns such as rigid cellular bridges, micropapillations or arcades should be classified as ADH or low-grade DCIS (Figure 4a–f). Similarly, high-grade cytologic atypia with nuclear pleomorphism of the type seen in high-grade DCIS is not a feature of lesions included in the CCL category; such lesions should be classified as high-grade DCIS.

Several authors have examined morphometric nuclear features in order to aid in the distinction of FEA from CCC. Using whole slide imaging and ImageJ software Katayama et al. compared morphologic parameters of 12 cases of CCC and FEA [60]. While cells in CCC had greater mean maximum Feret’s diameter (defined as the measure of an object size along a specified direction), major axis length and perimeter, cells in FEA had larger mean minimum Feret’s diameter, minor axis lengths and nuclear area. The nuclei of FEA also showed a significantly rounder shape as compared to nuclei of CCC. Similarly, in a cohort of 22 FEAs and 13 CCLs without atypia, Yamashita et al. used ellipticity as a nuclear parameter and also found that FEA has significantly rounder nuclei as compared to CCC/CCH [61]. These findings are in contrast to Lim et al. who reported that the Feret’s diameter, nuclear area and perimeter of FEA were significantly greater than in CCC whereas no difference was observed in circularity [62]. Overall, while nuclear morphology may distinguish FEA from CCC, further validation of morphometric features is required in order to determine which parameter is the optimal objective reference for diagnosis or in order to consider training artificial intelligence (AI)-based diagnostic algorithms.

Another point that merits consideration when discussing the histologic features of CCLs is their diagnostic reproducibility. Samples et al. assessed interobserver agreement of 29–30 pathologists for the diagnosis of six reference cases of FEA [63]. The rate of agreement with the reference diagnosis ranged from 17% to 52% with the reference panel pathologists demonstrating substantial variability in their interpretation of FEA. Tan et al. assessed the interobserver variability in a series of digitized images of CCLs presented to seven staff pathologists, and reported an intraobserver agreement of fair to substantial for the spectrum of CCLs (k = 0.334–0.669) [64]. The lowest number of complete agreements was achieved in the lesions characterized as CCC with cytologic atypia. When comparing the interobserver variability in the diagnoses of CCLs between general pathologists and a single pathologist with expertise in breast pathology, Gomes examined 610 breast specimens which were reviewed as part of a request for an external opinion (4.1% of these requests were generated by pathologists) [65]. There was weak diagnostic agreement between the original report and later review for CCC (k = 0.38); however, the agreement was moderate (k = 0.47) between the diagnoses of FEA.

Following training tutorials and with well-defined criteria for FEA, O’Malley et al. found an overall agreement of 91.8% for 30 cases of CCLs including complete agreement among all eight pathologists in 24 (80%) cases (k = 0.83) [66]. Darvishian’s group similarly found a kappa value of 0.85 when evaluating the reproducibility of FEA on CNB following a tutorial [67]. Haupt et al. analyzed the diagnostic agreement of CCL cases between residents and fellows before and after a tutorial on the diagnostic criteria of CCLs [68]. Before the tutorial the diagnostic agreement of FEA was weak (k = 0.39); however, after training there was a statistically significant increase in the ability to recognize FEA (k = 0.60). Therefore, this evidence suggests that interobserver variability may be improved following educational tutorials and using well-defined diagnostic criteria, although uniform classification of CCLs, especially FEA, may be challenging amongst pathologists in routine practice.

## 5. Cytologic Features

With the widespread use of CNB sampling, the cytologic features of CCLs are less relevant in the day-to-day work of practicing pathologists. Nevertheless, they have been described in several small cohorts. In a series of 20 fine needle aspirations (FNAs) with subsequent histologic diagnoses of CCLs, Saqi et al. described three-dimensional groups with overlapping cells, palisading columnar cells and occasional apocrine snouts [69]. Although 18 of 20 FNAs were described as atypical, only five of the cases demonstrated cytologic atypia on subsequent CNB. In another series of 10 cases of CCLs sampled by FNA followed by surgical excision, Jensen et al. describe sheets and papillary clusters of cells with well-delineated cell borders, round nuclei and finely granular cytoplasm with atypia ranging from mild to suspicious for malignancy [70]. Both studies note that due to substantial overlap with the cytologic features of other entities such as papillary lesions, fibroadenomas, malignant breast neoplasms and radiation changes, CCLs cannot be reliably diagnosed by FNA.

## 6. Immunohistochemical Markers

The immunohistochemical features of CCLs have been investigated in order to examine their relationship to other breast lesions and explore their etiology. Similar to other low-grade clonal proliferations such as ADH and low-grade DCIS, CCLs are usually diffusely positive for estrogen and progesterone receptors [7,11,23,24,29,71,72], cytokeratin 19 [7,11,29], Bcl-2 [73], and cyclin D1 [7]. High molecular weight cytokeratins (CK5/6, CK14) are largely negative [29,74] as is HER2 [75,76]. P53 expression has been found to parallel that of adjacent carcinoma [75].

Noel et al. investigated the proliferative rate in CCLs and found that the Ki-67 index was low (<1%) in CCC and CCH without atypia [77]. While the proliferative index was significantly higher (mean 8.2%) in FEA, it was lower than that of intermediate to high-grade DCIS (mean 25.4%). Paradoxically, the Ki-67 proliferative index in CCC and CCH without atypia was lower than in the adjacent TDLU (mean 2.4%), a finding postulated to be due to the fact that TDLUs are less sensitive to the effects of the menstrual cycle, age, oral contraceptive of hormonal replacement therapy, or because CCC without atypia represents only a simple transformation from cuboidal to tall columnar epithelium without modification of proliferative properties.

When examining a series of breast biopsies performed for mammographically detected calcifications, Fraser et al. described a spectrum of lesions in the TDLU characterized by columnar epithelial cells with prominent apical snouts, intraluminal secretions, varying degrees of cytologic and architectural atypia and associated calcifications which they termed “columnar alteration with prominent apical snouts and secretions (CAPSS)” [6]. Using CAPSS terminology, Dessauvagie et al. found Ki-67 immunostaining (9.16 ± 4.07%) increased as compared to normal breast (1.9 ± 0.67%) but lower than in situ and invasive lesions [72]. Similarly, Lee et al. found the Ki-67 proliferative index to be higher in CCLs designated “hyperplastic enlarged lobular unit (HELU)” (6.3 ± 0.47%) than in normal TDLUs (2.0 ± 0.42%), although there was no stratification based on the presence or absence of accompanying cytologic or architectural atypia [23]. Tomasino and colleagues investigated Ki-67 expression in biopsies with FEA/CCLs with “mild” atypia (FEA/CCHAm), “high” atypia (FEA/CCHAh) and normal breast tissue. The percentage of positive nuclei in normal tissue controls was 2.2 ± 0.50, 1.8 ± 0.60 in the CCHs, 3.0 ± 0.50 in the FEA/CCHAm, and 10.5 ± 1.40 in the FEA/CCHAh [78].

## 7. Molecular Alterations

Breast cancer is a diverse group of diseases with respect to morphologic features, clinical behavior and molecular profile. Low-grade invasive breast carcinomas frequently show recurrent chromosomal aberrations including loss of chromosome 16q and gain of 1q, while high-grade tumors are genetically complex lesions. Given the frequent coexistence of CCLs with other lesions in the low-grade breast neoplasia pathway, molecular studies have been employed to determine whether CCLs harbor similar genetic alterations that may support a clonal origin rather than a hyperplastic or proliferative process.

In the process of breast carcinogenesis, epigenetic abnormalities such as promoter CpG islands are considered to be an early event and may lead to inactivation of tumor suppressor genes, DNA repair genes, cell cycle regulators and transcription factors. Park et al. previously demonstrated that promoter CpG islands were significantly higher in FEA than in normal breast tissue and were similar in invasive carcinoma of no special type and DCIS [79]. Verschuur-Maes et al. also found significantly higher methylation levels of several tumor suppressor genes in CCLs as compared to normal breast tissue. They concluded that CpG island methylation of tumor-related genes is an early event in breast cancer progression and suggested that CCLs may act as precursors of breast cancer [80].

Further studies employing other molecular techniques such as loss of heterozygosity (LOH) [81,82], comparative genomic hybridization [29], in situ hybridization [83], microRNA (miRNA) in situ hybridization [84], mitochondrial DNA sequencing [82], multiplex ligation-dependent probe amplification [36], and allelic imbalance [85,86] have demonstrated that CCLs exhibit increasing genetic alterations as compared to normal breast epithelium with further alterations in DCIS and invasive carcinoma suggesting a step-wise progression. Similar chromosomal aberrations (especially 16q loss) may occur over the spectrum of FEA, ADH, low-grade DCIS, and low-grade invasive carcinoma of no special type.

Using the designation “clinging carcinoma in situ” with monomorphic features, Moinfar et al. investigated possible genetic alterations in 22 cases of pure flat lesions as well as cases with concurrent conventional DCIS and invasive carcinoma [81]. LOH was identified in 17/22 (77%) of cases at a minimum of one locus, including in 9 of 13 (70%) lesions of monomorphic type. In a subset of cases with concurrent DCIS or invasive carcinoma, identical LOH patterns were seen in flat lesions and adjacent DCIS or invasive carcinoma. The most common LOH loci in the flat lesions included 11q, 16q, and 3p. 

Using comparative genomic hybridization, Simpson et al. studied 81 lesions from 18 patients with CCLs co-existing with DCIS or invasive carcinoma of no special type [29]. All categories of CCLs exhibited a range of chromosomal copy number gains and losses and the recurrent changes included 16q, 17p or X loss, and 15q, 16p, and 19q gain. The genetic hallmarks of low-grade invasive carcinoma and DCIS were observed, namely, low numbers of chromosomal alterations, more frequent detection of losses relative to gains and recurrent loss of 16q. Lesions categorized as CCC with or without atypia showed a lower level of copy number changes relative to CCH and genetic complexity further increased with the presence of either cytologic or architectural atypia. 

Aulmann et al. sought to investigate the relationship of extensive FEA spanning greater than two TDLUs with associated low-grade DCIS using comparative allelotyping and loss of heterozygosity markers. Similar to localized FEA, in most cases the different foci of FEA and DCIS shared the same LOHs in most cases; however, in some cases, there were also areas of FEA without close relationship to the rest of the analyzed lesions suggesting a multifocal development [87].

Lastly, Verschuur-Maes et al. examined gene copy number of 17 breast cancer-related genes in CCLs, paired DCIS and invasive carcinoma using multiplex ligation-dependent probe amplification (MLPA) [88]. No high-level gene amplifications were observed in CCLs but copy number gains in *C11orf30*, *MYC*, *CPD*, *MTDH*, *CCND1*, *CCNE1*, *ESR1*, and *TOP2A* genes were encountered. The frequency of gene copy number changes increased from CCL towards DCIS and invasive carcinoma. The authors conclude that CCLs carry copy number changes of several known breast cancer-related genes, thereby substantiating their role in breast carcinogenesis.

Overall, these molecular observations demonstrate genetic commonality between CCLs and other members of the low-grade breast neoplasia pathway supporting the notion that CCLs are clonal precursor lesions. Further studies are required to elucidate factors that determine the rate of progression to in situ and invasive malignancy.

## 8. Columnar Cell Lesions in the Male Breast

Several studies have assessed the presence of CCLs in the male breast. Ni et al. examined consecutively excised male breast specimens and described “columnar cell-like” lesions composed of dilated ducts with intact surrounding myoepithelial cells and an inner layer of columnar luminal cells and apical snouts in 54% of cases [89]. Similar to CCLs in the female breast, most of the columnar cells were negative for CK5/6 and positive for estrogen receptor with moderate to strong intensity in the majority of cells. As the “columnar cell-like” lesions lacked some of the typical morphologic features of CCLs seen in women (mainly intraluminal secretions and calcifications), the authors questioned whether these lesions represent genuine counterparts to CCLs in men. Verschuur-Maes et al. subsequently examined all male breast cancer resection specimens (including invasive carcinoma and DCIS) over a 24 year period as well as 20 gynecomastia cases and five clinically normal male breasts sampled at autopsy [90]. While they identified apical snouts on the luminal border in some ducts, no typical CCL features were seen such as cystically dilated acini, secretions or calcifications. Morphologic and immunohistochemical features supportive of gynecomastia were also noted [91]. The authors stated that while the presence of CCLS cannot be completely excluded, they seem to be uncommon and are therefore unlikely to play a major role in male breast carcinogenesis. Of note, both aforementioned studies lacked additional molecular analyses to evaluate genomic aberrations. 

Lastly, in a retrospective joint analysis of the International Male Breast Cancer Program central pathology review was performed for 1328 male breast cancer patients [92]. Although pathology review was limited to one representative formalin-fixed-paraffin-embedded tissue block per case, a columnar cell-like lesion was detected in 13 patients, of which 11 also had adjacent DCIS. Next generation sequencing was performed on three of these cases and similar mutations (including *PIK3CA* and *GATA3*) were found in the CCLs and the adjacent invasive carcinoma in 2/3 patients. This small study concluded that despite the low prevalence of CCLs in the male breast, the findings support the hypothesis that CCLs are a putative precursor lesion in male patients. 

## 9. Clinical Significance

CCLs are commonly identified in association with lobular carcinoma in situ or atypical lobular hyperplasia, low-grade DCIS and tubular carcinoma [6,7,8,9,10,11,12,13,14,15,16,17,18,19,20]. Although morphologic, immunohistochemical and molecular evidence supports that FEA is a non-obligate precursor to low-grade DCIS and invasive carcinoma, the clinical significance of isolated CCLs as well as their appropriate management when diagnosed on CNB remain controversial. The challenge in elucidating the clinical significance of CCLs has in part been secondary to the heterogeneity of terms used to describe CCLs in the medical literature as well as the small sample size in early observational series. Further challenges include the variation in radiologic-pathologic correlation, biopsy sampling techniques, the variation in the removal of radiologically targeted calcifications at biopsy, as well as the presence or absence of pathology review. 

In regards to overall breast cancer risk associated with CCLs, Boulos et al. evaluated the cancer risk for 1261 biopsies with CCLs [93]. They observed that the presence of CCL alone was associated with a mild increase in overall breast cancer risk (relative risk 1.47, 95% CI, 1.0–2.2). Other authors have found that FEA does not convey an independent risk of breast cancer beyond that of associated proliferative disease without atypia or associated atypical hyperplasia [5,94]. In a study of 9000 breast biopsies, 25 patients were found to have pure FEA, none of which underwent excision [95]. After an average follow-up of 19.2 years, one patient (4%) had recurrent or persistent FEA, while none developed breast cancer. Additional studies found one or no recurrences in cases of pure FEA with 5.4 and 10 years of follow-up [96,97]. Therefore, although FEA may convey a mildly increased risk in breast cancer, the overall risk of progression is low and may not be independent of any coexisting proliferative lesions.

As suggested by Drs. S. Schnitt and A. Vincent-Salomom, when CCC or CCH are encountered in a CNB specimen, no additional work-up or excision is required [28]. In contrast, when pure FEA is encountered in CNB, the optimal management is less clear due to a wide range of reported upgrade rates. The reported incidence of pure FEA diagnosed on CNB varies from <1–22% [9,98]. Similarly, the reported rate of upgrade to malignancy ranges from 0% to 38% [8,9,10,32,38,39,40,41,42,43,46,49,51,67,98,99,100,101,102,103,104,105,106,107,108,109,110,111,112,113,114,115,116,117,118,119,120,121,122,123,124,125,126,127]. Some studies have suggested criteria for excision, including assessment of radiologic features (i.e., mass versus calcifications) [50], span of calcifications [38] residual calcifications post CNB [103,111,117], and consideration of personal history of breast cancer [117]. Other studies recommend performing additional deeper levels on any CNB that displays FEA as FEA may evolve into ADH within the same focus [100].

In order to provide a more robust evaluation, several recent systemic reviews and meta-analyses have been performed in order to identify the pooled upgrade rates of pure FEA to malignancy. Wahab et al. reviewed 2482 cases of pure FEA over 42 studies published from 2004–2020 managed either by surgical excision or imaging follow-up [128]. Their analysis demonstrated a pooled upgrade rate of 5% to all breast cancers, 1% to invasive carcinoma and 2% to DCIS. When >90% of targeted calcifications were removed, the pooled upgrade rate to breast cancer decreased to 0%. In this case, 16 studies in the pooled analysis described criteria for management with surgical excision versus close imaging follow-up, 17 recommended surgical excision, six studies recommended close imaging follow-up and three studies made no management recommendations. Similarly, another systemic review and meta-analysis examined 1924 cases of pure FEA among 59 studies managed with surgical excision [129]. The overall pooled upgrade rate to malignancy was 8.8%. Lastly, Rudin et al. reviewed 1966 cases of pure FEA, 77% of which underwent surgical excision [130]. The pooled estimate of upgrade to cancer was 11.1%. When restricted to “16 higher-quality studies” the pooled upgrade estimate was 7.5%. In all aforementioned meta-analyses, the authors support the excision of pure FEA rather than imaging follow-up; however, one may choose to follow Wahab’s group’s recommendation stating that if >90% of targeted calcifications are removed, close imaging follow-up could be performed instead of surgical excision.

Given the lack of consensus recommendations for the management of FEA, it is not surprising that the approach to their management is heterogeneous. In an audience response survey at the 2010 and 2011 annual meetings of the American Roentgen Ray Society, radiologists were asked about the clinical management of various high-risk lesions diagnosed on CNB [131]. In the 2010 respondent cohort, 73% indicated they would recommend surgical excision of FEA, while in 2011 this number rose to 82%. In a survey of breast imagers at 41 academic institutions in the United States in 2016–2017, Falomo et al. examined the management recommendations for high-risk lesions diagnosed on CNB [132]. Surgical excision was the reported recommendation from 76% of respondents for pure FEA, 7% recommended returning to screening, 2% recommended short interval follow-up, and 15% responded that management was dependent on certain factors. The authors also noted that in institutions from which multiple surveys were received, 86% had conflicting responses for at least one management recommendation suggesting variability amongst colleagues. Kappel et al. surveyed members of the Canadian Society of Surgical Oncology, Canadian Association of General Surgeons and Canadian Association of Radiologists with regards to practice recommendations in high-risk benign breast disease [133]. The 41 respondents (19 surgeons and 22 radiologists) were unable to reach consensus for any of the five high-risk lesion scenarios. In regards to FEA, 46% of respondents recommended excision, while 49% recommended imaging follow-up. The remaining 5% interpreted the clinical scenario findings as discordant and recommended either re-biopsy or surgical excision. 

If one turns to national and international societies, guidelines for the management of FEA are scant. The American Society of Breast Surgeons currently recommends observation with clinical and imaging follow-up if pure FEA or CCL is diagnosed on CNB but excision in the presence of concurrent ADH [134]. As for Europe, the Second International Consensus Conference on lesions of uncertain malignant potential in the breast (B3 lesions) recommends minimally invasive management of FEA diagnosed on CNB with vacuum-assisted biopsy (VACB) [135]. If FEA is thereafter diagnosed on VACB, surveillance rather than surgical excision is recommended. The United Kingdom National Health Service Breast Screening Programme (NHSBP) guidelines state that if FEA is diagnosed on CNB or VACB, multidisciplinary discussion is warranted, followed by vacuum-assisted excision (VAE) [136]. If no additional atypia is present following VAE, a return to annual screening is suggested.

The latest edition of the WHO classification of tumors of the breast states that the need for routine surgical excision after a diagnosis of FEA on CNB is uncertain. Furthermore, surgical excision may not be necessary if a post-biopsy mammogram shows that all of the radiographic lesion has been removed. Radiologic-pathologic correlation is recommended for guiding further management [1].

## 10. Conclusions

Immunohistochemical and molecular studies suggest that CCLs represent the earliest non-obligate precursor of low-grade breast carcinoma. Nevertheless, the risk of local recurrence and progression to carcinoma is low. Management recommendations when pure FEA is diagnosed on CNB are heterogeneous, although recent meta-analyses suggest a pooled upgrade rate of at least 5%. 

## Data Availability

Not applicable.
